# Beyond traditional roles: vitamin D and erythropoietin as immune modulators in kidney diseases

**DOI:** 10.1093/ckj/sfag120

**Published:** 2026-04-28

**Authors:** Lorenza Magagnoli, Sofia Bin, Paolo Cravedi, Mario Cozzolino

**Affiliations:** Department of Health Sciences, University of Milan, Milano, Italy; Renal Division, Azienda Socio-Sanitaria Territoriale (ASST) Santi Paolo e Carlo, Milano, Italy; Department of Medical and Surgical Sciences (DIMEC), Alma Mater Studiorum–University of Bologna, Bologna, Italy; Nephrology, Dialysis and Kidney Transplant Unit, IRCCS Azienda Ospedaliero, University of Bologna, Bologna, Italy; Translational Transplant Research Center, Department of Medicine, Icahn School of Medicine at Mount Sinai, New York, NY, USA; Department of Health Sciences, University of Milan, Milano, Italy; Renal Division, Azienda Socio-Sanitaria Territoriale (ASST) Santi Paolo e Carlo, Milano, Italy

**Keywords:** EPO, immunity, kidney disease, transplant, vitamin D

## Abstract

Vitamin D and erythropoietin (EPO) are kidney-derived hormones classically known for their roles in mineral metabolism and erythropoiesis, respectively. Beyond these functions, growing evidence indicates that both molecules exert broad immunomodulatory effects on innate and adaptive immunity. Vitamin D signalling through the vitamin D receptor shapes dendritic cell maturation, promotes regulatory T-cell induction, and suppresses pro-inflammatory T helper cell responses. Similarly, EPO acts as a pleiotropic cytokine capable of modulating macrophage activation, T-cell proliferation, and inflammatory signalling pathways through EPO receptor-dependent mechanisms.

In chronic kidney disease (CKD), reduced renal synthesis of active vitamin D and impaired endogenous EPO production frequently coexist, contributing not only to disturbances in mineral metabolism and anaemia, but possibly also to immune dysregulation. Besides CKD, immune dysregulation is common across diverse nephrological conditions, including immune-mediated nephropathies and transplantation, where inflammatory and alloimmune responses critically influence disease progression and graft outcomes. Increasing experimental and clinical evidence suggests that vitamin D and EPO may modulate these processes and represent potential therapeutic targets.

This narrative review summarizes current knowledge on the immunomodulatory properties of vitamin D and EPO, their mechanisms of action on immune cells, and their relevance in kidney disease and transplantation.

## INTRODUCTION

Not all organs are created immunologically equal. Experimental studies in mice, pigs, and nonhuman primates have consistently demonstrated that kidney allografts display unique immune properties compared with other transplanted organs. In several models, kidney grafts show an intrinsic capacity to modulate alloimmune responses and, under specific conditions, promote systemic immune regulation [1]. Although the cellular and molecular mechanisms underlying this distinctive immunological behaviour remain incompletely defined, increasing evidence points to kidney-derived hormones, particularly vitamin D and erythropoietin (EPO), as potential contributors to immune regulation.

Beyond their classical roles in mineral and bone metabolism and erythropoiesis, respectively, vitamin D and EPO exert broad immunomodulatory effects on both innate and adaptive immune cells. Vitamin D shapes dendritic cell (DC) maturation, promotes regulatory T-cell (Treg) induction, and restrains pro-inflammatory cytokine production [2]. EPO is now recognized as a pleiotropic cytokine capable of modulating macrophage activation, T-cell responses, and inflammatory signalling pathways [3]. Together, these pathways may contribute to the kidney’s distinctive immunological profile.

Importantly, the immunoregulatory properties of vitamin D and EPO are increasingly being tested in clinical trials in autoimmune diseases and transplant rejection, whereas their inhibition has been proposed for the treatment of cancers where EPO-mediated immune suppression may favour tumour progression. A deeper understanding of their immune modulatory roles is therefore of direct translational relevance.

This review does not focus on the well-known endocrine roles of vitamin D and EPO. In contrast, we examine current knowledge regarding the immunomodulatory effects of vitamin D and EPO in physiological conditions and in kidney disease, with particular emphasis on their implications for transplantation and immune-mediated pathology. Literature was identified through searches of PubMed and Scopus reports published from 2000 to 31 January 2026 using combinations of the terms ‘vitamin D/cholecalciferol/ergocalciferol/calcifediol/calcitriol’, ‘erythropoietin/EPO’, ‘kidney/renal disease’, ‘immune/immunity’, and ‘transplant/transplantation’. We included mechanistic, preclinical, and clinical studies, prioritizing recent and high-impact publications. Given the narrative nature of this review, no formal systematic selection process or quality assessment was performed, and the potential for selection bias should be acknowledged.

## VITAMIN D

### Vitamin D physiology, receptor signalling, and dysregulation in CKD

Vitamin D metabolism involves two essential hydroxylation steps required for the generation of the active form, 1,25(OH)_2_D. The first occurs in the liver, where either cholecalciferol (D_3_) or ergocalciferol (D_2_) is converted into 25-hydroxyvitamin D [25(OH)D], the main circulating form. The second and biologically critical hydroxylation primarily takes place in the renal proximal tubules, where the enzyme 1α-hydroxylase converts 25(OH)D into 1,25(OH)_2_D [5].

This active molecule exerts its biological effects primarily through binding to the intracellular vitamin D receptor (VDR), a transcription factor belonging to the nuclear receptor superfamily [6]. The VDR is expressed in a wide range of tissues, including organs directly involved in calcium–phosphate homeostasis (e.g. intestine, bone, and kidney) as well as other sites that are not primarily engaged in mineral metabolism. Upon ligand binding, the VDR heterodimerizes with the retinoid X receptor and translocates into the nucleus, where it interacts with vitamin D response elements in target genes promoters to regulate transcription of hundreds of genes involved in mineral metabolism, as well as cellular proliferation, differentiation, and immune function. In addition to these genomic effects, rapid nongenomic actions mediated by membrane-expressed VDR have also been described [7]. These involve activation of second-messenger systems and kinase cascades, including phospholipase C, protein kinase C, mitogen-activated protein kinases (MAPKs), and phosphatidylinositol 3-kinase (PI3K)/Akt signalling, enabling rapid modulation of cellular responses independently of gene transcription. In chronic kidney disease (CKD), the renal activation of vitamin D is impaired, as the progressive loss of functioning nephron mass reduces the number of proximal tubular cells expressing 1α-hydroxylase. Elevated fibroblast growth factor 23, a hallmark of CKD, further suppresses 1α-hydroxylase while simultaneously inducing 24-hydroxylase, the enzyme responsible for catabolizing both 25(OH)D and 1,25(OH)_2_D [8]. Systemic inflammation—commonly observed in CKD fuels persistently high levels of tumour necrosis factor alpha (TNF-α) and interleukin-6 (IL-6), which also downregulate renal 1α-hydroxylase expression [9].

In parallel, CKD is associated with reduced levels not only of the active metabolite 1,25(OH)_2_D but also of its precursor 25(OH)D. Together, these mechanisms contribute to reduced circulating levels of both 25(OH)D and 1,25(OH)_2_D in CKD. Moreover, VDR expression may be reduced in CKD due to uraemia, inflammation, and secondary hyperparathyroidism, potentially contributing to vitamin D resistance and impaired downstream signalling [12].

### Effects of vitamin D on innate immunity

**Figure 1:**
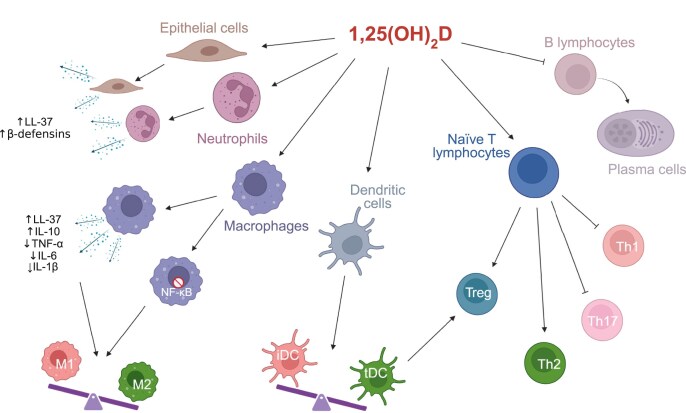
Immunomodulatory pathways of vitamin D. The active vitamin D metabolite 1,25(OH)_2_D exerts broad effects on innate and adaptive immunity. In innate immune cells, vitamin D enhances antimicrobial defence by increasing the production of antimicrobial peptides in epithelial cells, neutrophils, and macrophages, while promoting an anti-inflammatory macrophage phenotype (M2 polarization) and inhibiting NF-κB-driven pro-inflammatory cytokine production. Vitamin D also impairs DC maturation, favouring the development of tolerogenic (tDC) rather than immunogenic phenotype (iDC), that promote Treg induction. In adaptive immunity, vitamin D modulates T-cell differentiation by suppressing Th1 and Th17 responses while promoting Th2 and Tregs, thereby contributing to immune tolerance. Additionally, vitamin D inhibits B-cell differentiation into plasma cells. Created using BioRender.

Both 1α-hydroxylase and the VDR are expressed in multiple immune cells, including macrophages, DCs, and lymphocytes. This enables them to locally convert circulating 25(OH)D into active 1,25(OH)_2_D, allowing for autocrine and paracrine signalling within immune tissues independent of renal function [13]. The capacity of immune cells to autonomously regulate vitamin D activation provides a mechanistic basis for its broad immunomodulatory effects across both innate and adaptive immune pathways [14], as illustrated in Fig. 1.

A major mechanism by which vitamin D enhances innate defence is through VDR-mediated upregulation of some antimicrobial peptides, such as cathelicidin (LL-37) and β-defensins, in monocytes, macrophages, neutrophils, and epithelial cells [15]. These molecules are key components of the innate immune barrier: LL-37 disrupts microbial membranes and neutralizes endotoxins, whereas β-defensins act as chemotactic and microbicidal peptides at epithelial and mucosal surfaces, providing broad antibacterial, antiviral, and antifungal protection [15, 16].

Local production of 1,25(OH)_2_D further enhances macrophage function by promoting phagocytosis, microbial killing, and by modulating cytokine production. Specifically, VDR activation suppresses the transcription of pro-inflammatory cytokines, such as TNF-α, interferon-γ (IFN-γ), and IL-6, while promoting the expression of anti-inflammatory mediators including interleukin-10 (IL-10) [17]. This contributes to a shift in macrophages from a classically activated (M1) to an alternatively activated (M2) phenotype, at least in part through activation of Janus kinase (JAK)/signal transducer and activator of transcription 3 (STAT3) signalling pathways, and downstream metabolic regulators such as Peroxisome Proliferator-Activated Receptors (PPARγ), which collectively favour anti-inflammatory polarization, associated with tissue repair and resolution of inflammation [18, 19].

In addition to these transcriptionally-induced effects, vitamin D also modulates innate immune activation through rapid mechanisms that are not mediated by changes in gene transcriptome. Liganded VDR directly interacts with IκB kinase (IKKβ), a key kinase in the nuclear factor kappa-light-chain-enhancer (NF-κB) pathway, thereby disrupting IKK complex assembly and preventing IκBα phosphorylation and degradation. This stabilizes IκBα and blocks NF-κB nuclear translocation, leading to reduced expression of pro-inflammatory mediators [20]. Given the central role of NF-κB in driving M1 macrophage polarization, this VDR-IKKβ-dependent mechanism may also contribute to the shift towards an anti-inflammatory M2 phenotype [18].

Vitamin D also exerts important effects on DCs. Exposure to 1,25(OH)_2_D impairs DC maturation, through a reduced expression of surface costimulatory molecules (CD80, CD86, and Major Histocompatibility Complex (MHC) class II. C) and decreased secretion of pro-inflammatory cytokines such as interleukin-12 (IL-12) [21]. These changes result in the development of a tolerogenic DC phenotype (tDC), which, upon antigen encounter, favours the induction of Tregs over effector T cells, thereby promoting an anti-inflammatory response [22, 23].

### Effects of vitamin D on adaptive immunity

Vitamin D also affects adaptive immunity by modulating T and B lymphocytes differentiation and function (Fig. 1).

Experimental studies suggested that vitamin D inhibits Th1 and Th17 responses while promoting Th2 and Tregs function. 1,25(OH)_2_D suppresses the *in vitro* differentiation of naїve T cells into Th1 cells and reduces their production of IFN-γ [24]. Similarly, it downregulates interleukin-17 (IL-17) synthesis by Th17 cells, and experimental autoimmune encephalomyelitis in mice, a Th17-mediated condition [25]. In contrast, vitamin D promotes the *in vitro* conversion of naїve T cells into Tregs and Th2 cells, through direct effects on T cells, in addition to the DC-mediated mechanism described above. Specifically, 1,25(OH)_2_D enhances Th2 polarization through increased interleukin-4 (IL-4) production and supporting Tregs differentiation via upregulation of transforming growth factor-β (TGF-β), IL-10, and the transcription factor FOXP3 [26, 27]. These shift towards Tregs and Th2 cells promote an anti-inflammatory immune profile and favour immune tolerance.

Collectively, this vitamin D-driven immunologic shift towards a more tolerogenic and anti-inflammatory T-cell profile may be particularly relevant in autoimmune and inflammatory diseases. However, whether these biological mechanisms translate into clinically meaningful effects of vitamin D supplementation in humans with autoimmunity remains uncertain. The few available trials exploring the effect of vitamin D supplementation on T-cell subpopulations and cytokines levels in patients with autoimmune diseases—including systemic lupus erythematosus (SLE), Hashimoto thyroiditis, and multiple sclerosis—have yielded inconsistent results [28–31]. In contrast, randomized controlled trials in type 1 diabetes mellitus have shown that cholecalciferol supplementation can increase both the number and suppressive function of Tregs, supporting a potential disease-specific immunomodulatory effect [32].

Vitamin D also inhibits the *in vitro* differentiation of B cells into plasma cells and reduces memory B cell formation, thereby indirectly limiting immunoglobulin production and potentially decreasing the generation of pathogenic autoantibodies [33, 34]. However, *in vivo* evidence on the effects of vitamin D on B cell function remains limited. In patients with multiple sclerosis, short-term high-dose cholecalciferol supplementation did not alter B cell differentiation or serum immunoglobulin levels [35]. Conversely, in patients with SLE, high-dose vitamin D reduced class-switched memory B cells and anti-double-stranded DNA (anti-dsDNA) autoantibodies, suggesting that vitamin D may preferentially affect antigen-specific responses in conditions of ongoing immune activation [31]. This selectivity aligns with the concept that short-lived autoreactive plasma cells—generated continuously in autoimmune diseases—may be more susceptible to vitamin D–mediated regulation than long-lived plasma cells.

### Immunomodulating effects of vitamin D in kidney disease and transplantation

The immunomodulatory mechanisms described above may have relevant implications across several nephrological conditions, including immune-mediated nephropathies, CKD, and kidney transplantation.

In immune-mediated kidney diseases, observational studies consistently report an inverse association between serum 25(OH)D levels and disease activity, independent of kidney function [36–39]. Moreover, low 25(OH)D concentrations have been linked to an increased risk of developing lupus nephritis among patients with SLE [37, 38], as well as to more severe histopathological features in IgA nephropathy [40]. Notably, treatment with vitamin D analogues has proven effective in reducing proteinuria and autoantibody levels in animal models of lupus nephritis, Heyman nephritis (a rat model to study membranous nephropathy), and mercuric chloride-induced autoimmune glomerulonephritis [41].

Chronic low-grade inflammation is integral to CKD pathogenesis and cardiovascular risk, driven by oxidative stress, uremic toxins, immune dysregulation, and gut-derived endotoxins [4]. In this context, chronic inflammation and immune dysfunction contribute to kidney disease progression, malnutrition, anaemia, mineral disorders, atherosclerosis, and increased susceptibility to infections [4, 42–44], thereby representing potential therapeutic targets. A limited number of small randomized controlled trials have evaluated the anti-inflammatory effects of vitamin D analogues in patients with CKD, with inconsistent results [45–55] (Table 1). Despite the supportive findings, these studies are characterized by substantial heterogeneity in design, populations, and interventions, and have frequently reported neutral or only modest effects on systemic inflammatory markers. Moreover, evidence supporting a beneficial impact on hard clinical outcomes remains limited.

**Table 1:** Randomized controlled trials evaluating the immunological effects of vitamin D analogues in patients with CKD.

Author, year	Population	Treatments	Duration	Endpoints related to inflammation or immunomodulation	Results related to inflammation or immunomodulation
Moe SM, 2001	*N* = 31;MHD;USA	Paricalcitol 4 µg three times weeklyvs placebo	12 weeks	*Ex vivo*:- Antigen-specific PBMC proliferation; - Mitogen-induced PBMC proliferation;- PBMC cytokine production (IL-6, IL-2, TNF-α, IFN-γ);*In vivo*:- Delayed-type hypersensitivity skin testing to purified protein derivative, mumps, *Candida, Trichophytin*; - Anti-hepatitis B antibody titer following a booster vaccination;- Number of infections.	- Anergic patients treated with paricalcitol have a tendency towards improving delayed hypersensitivity reactions (4 of 11 patients in the paricalcitol group vs 0 of 9 patients in the placebo group converted to reactive, *p* = 0.09);**—** no significant differences in all other outcomes.
Alborzi P, 2008	*N* = 24;CKD with eGFR ≥30 ml/min on a stable dose of RAASi;USA	Paricalcitol 1 µg/dayvsparicalcitol 2 µg/dayvsplacebo	4 weeks	Changes in high-sensitivity CRP (hsCRP)	Changes in hsCRP levels differed among treatments (*P* .048):- In patients treated with paricalcitol 1 µg/day hsCRP decreased from 2.4 (1.2, 4.5) mg/dl to 1.9 (0.6, 6.4) mg/dl, *P* .62;- In patients treated with paricalcitol 2 µg/day hsCRP decreased from 4.3 (1.7, 11) mg/dl to 2.3 (0.9, 6.2) mg/dl, *P* .03;- In patients treated with placebo hsCRP increased from 5.4 (2.2, 13.1) mg/dl to 6.1 (3, 12.4) mg/dl, *P* .02.
Marckmann P, 2012	*N* = 52;CKD G3-5D;Denmark	Cholecalciferol 50.000 IU weekly vs placebo	8 weeks	Changes in IL-6 and CRP	No significant changes in IL-6 and CRP levels in any treatment, nor significant differences between treatments.
Alvarez JA, 2013	*N* = 46; CKD G2-3;veterans (91% males);USA	Cholecalciferol 50 000 IU weekly for 12 weeks followed by 50 000 IU every other week for 40 weeksvs matching placebo	52 weeks	Changes in TNF-α, IL-6, MCP-1, CXCL10, and NGAL	Changes in MCP-1 by week 12 differed between treatments (*P* .02):- In patients treated with cholecalciferol MCP-1 decreased by −6.2 ± 13.3 pg/ml from baseline value of 68.1 ± 16.7 pg/ml;- In patients treated with placebo MCP-1 increased by 6.5 ± 14.2 pg/ml from baseline value of 61.5 ± 8.7 pg/ml;However, no significant difference between treatments at week 52. No significant changes in TNF-α, IL-6, CXCL10, and NGAL, nor significant differences between treatments.
Piñera-Haces C, 2013	*N* = 26;MHD;Spain	Paricalcitol 1 µg/dayvs calcifediol 0266 µg/week	3 months with single treatments +3 months of combined treatments (paricalcitol and calcifediol in both groups)	Changes in IL-8	IL-8 levels decreased with both treatments:- In patients treated with paricalcitol IL-8 decreased from 856 ± 2.10 pg/ml at baseline to 73.7 ± 85 pg/ml after 3 months; *P* < .001;- In patients treated with calcifediol IL-8 decreased from 1168 ± 2.42 pg/ml at baseline to 43.8 ± 36 pg/ml after 3 months; *P* < .001.The difference between treatments was not tested.The combination of both treatments did not produce additional decreases.
Thethi TK,2015	*N* = 60;CKD G3-4 and T2DM;USA	Paricalcitol 1 µg/dayvs placebo	3 months	Changes in IL-6, hsCRP, urinary isoprostane, MCP-1, ICAM-1	No significant changes in IL-6, urinary isoprostane, MCP-1, ICAM-1 in any treatment, nor significant differences between treatments.In patients treated with placebo, hsCRP increased from 4.3 (0.2–13.1) mg/ml at baseline to 6.9 (0.8–13) mg/ml after 3 months, *P* .02; while did not change in patients treated with paricalcitol.
Meireles MS, 2016	*N* = 38 HD; MHD or PD and 25(OH)D levels <20 ng/ml;BrazilHemoa	Cholecalciferol 50.000 IU twice weekly vsplacebo	12 weeks	Changes in:- Monocyte expression of VDR, CYP27B1, CYP24A1 and IL-6; - Serum levels of IL-6, TNF-α and CRP.	In monocytes:- In patients treated with cholecalciferol CYP27B1 and VDR expression increased (*P* < .05), whereas IL-6 and CYP24A1 expression did not change;- In patients treated with placebo VDR expression decreased (*P* < .05), whereas IL-6, CYP27B1, and CYP24A1 expression did not change;- Differences between groups were not tested.Significant differences in changes of serum IL-6 and CRP levels between treatments (*P* < .05):- In patients treated with cholecalciferol IL-6 levels decreased from 8.1 ± 6.6 pg/ml to 4.6 ± 4.1 pg/ml (*P* < .05); CRP levels decreased from 0.50 (0.10–1.27) mg/dl to 0.28 (0.09–0.62) mg/dl (*P* < .05); TNF-α levels did not change;- In patients treated with placebo no significant changes in any inflammatory marker.
Kendrick J, 2017	*N* = 128;CKD G3b-4 and 25(OH)D levels <30 ng/ml;USA	Cholecalciferol 4000 IU/day for 1 month followed by 2000 IU/day for 5 monthsvs calcitriol 0.25 µg/day for 1 month followed by 0.25 or 0.5 µg/day depending on the calcium levels for 5 months	6 months	Changes in:-Serum levels of CRP and IL-6;-Endothelial cell expression of NFκB.	No significant changes in any treatment, nor significant differences between treatments.
Mansouri L, 2017	*N* = 36; CKD G3-4;Sweden	Paricalcitol 1 µg/dayvsparicalcitol 2 µg/dayvsplacebo	12 weeks	Changes in:- Plasma levels of IL-1β, IL-2, IL-4, IL-5, IL-6, IL-7, IL-8, IL-9, IL-10, IL-12, IL-13, IL-15, IL-17a, TNF-α, IFN-γ, MCP-1, CXCL10, MIP-1α and β, eotaxin, CCL5, G-CSF, GM-CSF, b-FGF, VEGF, and PDGF;- Plasma levels of several microRNAs (Exiqon miRCURY Ready-to-Use PCR Human panel I V1.M).	Among several cytokines:- In patients treated with paricalcitol 1 µg/day, VEGF decreased from 20 (15–31) pg/ml to ໿10 (3–15) pg/ml, *P* .01; PDGF decreased from 1297 (960–1933) pg/ml to 784 (561–915) pg/ml, *P* .005; the levels of other cytokines did not change;- In patients treated with paricalcitol 2 µg/day, VEGF decreased from 26 (9–60) pg/ml to ໿10 (7–32) pg/ml, *P* .02; PDGF decreased from 2811 (1447–3706) pg/ml to 1234 (1003–2239) pg/ml, *P* .009; CXCL10 decreased from 2628 (511–6200) pg/ml to 492.5 (270–2022) pg/ml, *P* .02; the levels of other cytokines did not change;- In patients treated with placebo no significant changes in any marker;- Differences between treatments were not tested. Among selected microRNAs, miR 432–5p, miR 495–3p, and miR 576–5p were significantly downregulated in the active treated groups, compared to the placebo group.
Nata N, 2022	*N* = 70;MHD and 25(OH)D levels <30 ng/ml;Thailand	Ergocalciferol ‘conventional dose’ [50.000 IU weekly or monthly base on 25(OH)D levels]vs ergocalciferol ‘high dose’ [100.000 IU weekly or monthly base on 25(OH)D levels]	8 weeks	Changes in IL-6	No significant changes in any treatment, nor significant differences between treatments in the overall population. However, among patients with baseline 25(OH)D levels <20 ng/ml, those treated with the high dose had a significant decline in IL-6 of −2.67 [−6.56, −0.17] pg/ml, *P* = .04.
Nugroho P, 2025	*N* = 120;DKD (defined as presence of T2DM and UACR > 30 mg/g) with eGFR >45 ml/min;Indonesia	Calcitriol 0.25 µg/day vsplacebo	6 months	Changes in IL-6	Significant difference between treatments (*P* .006):- In patients treated with calcitriol IL-6 levels did not change;- In patients treated with placebo IL-6 levels increased from 1.03 [0.35, 3.35] pg/ml to 2.94 [1.13, 9.92] pg/ml.

b-FGF, basic fibroblast growth factor; CCL5, C-C motif chemokine ligand 5; CRP, C-reactive protein; CXCL10, C-X-C motif chemokine 10; CYP24A1, cytochrome P450 family 24 subfamily A member 1; CYP27B1, cytochrome P450 family 27 subfamily B member 1; DKD, diabetic kidney disease; eGFR, estimated glomerular filtration rate; G-CSF, granulocyte colony-stimulating factor; GM-CSF, granulocyte monocyte colony-stimulating factor; ICAM-1, intercellular adhesion molecule 1; MIP, macrophage inflammatory protein; MHD, maintenance hemodialysis; *N*, number of participants; NGAL, neutrophil gelatinase-associated lipocalin; PBMC, peripheral-blood mononuclear cells; PDGF, platelet-derived growth factor; RAASi, renin–angiotensin–aldosterone system inhibitor; T2DM, type 2 diabetes mellitus; UACR, urinary albumin-to-creatinine ratio; VEGF, vascular endothelial growth factor; HD, Hemodialysis; PD, Peritoneal Dialysis.

In kidney transplantation, observational studies have highlighted that vitamin D deficiency is common and linked to worse graft function, increased rejection risk, higher incidence of proteinuria [56], the development of donor-specific antibodies [57], and increased infection-related mortality [58]. Evidence from experimental transplantation models in other organs supports a potential immunomodulatory role of vitamin D. In a rat lung transplantation model, calcitriol reduced acute cellular rejection and attenuated alloimmune responses [59]. Extensive data also derive from syngeneic and allogeneic islet transplantation models, in which calcitriol and vitamin D analogues—particularly when combined with conventional immunosuppressive agents such as cyclosporine A, mycophenolate mofetil, or anti-CD3 antibodies—prolonged graft survival, reduced inflammatory cytokine expression, limited immune cell infiltration, and promoted Treg responses [60]. These effects appear to be mediated by a shift from Th1- towards Th2-dominant immunity, induction of tDCs, and suppression of macrophage recruitment and NF-κB-dependent inflammatory pathways. Despite these promising experimental findings, clinical evidence remains scarce. A randomized trial in lung transplant recipients did not demonstrate a reduction in rejection rates with high-dose cholecalciferol supplementation [61], and robust clinical studies in other transplant populations are lacking.

Overall, current data suggest an immunomodulatory role for vitamin D in immune-mediated nephropathies, CKD, and transplantation, but definitive evidence of clinical benefit is still insufficient. Most associations come from observational studies and should be interpreted with caution, as vitamin D deficiency may reflect disease severity, reduced sun exposure, nutritional status, or immunosuppressive burden rather than a direct causal relationship.

## ERYTHROPOIETIN

### EPO physiology, receptor signalling, and dysregulation in CKD

EPO is a 34-kDa glycoprotein hormone produced mainly by foetal liver cells and, in adult life, by pericapillary interstitial fibroblasts of the kidney, in response to hypoxia [62]. Its main physiological function is the regulation of erythropoiesis, through the promotion of erythroid progenitor cell survival, proliferation, and differentiation [63, 64]. Beyond its hematopoietic function, EPO also acts as a cytokine and growth factor, affecting multiple organs and the immune system [3, 65–67].

On erythroid progenitor cells, the EPO receptor (EPOR) exists as a preformed homodimer that requires only low (picomolar) EPO concentrations for activation [68]. Ligand binding induces conformational changes that initiate intracellular signalling via JAK2, MAPK, PI3K, and STAT5, ultimately leading to increased red blood cell mass and improved oxygen delivery [69]. In nonerythroid cells, including immune cells, EPOR can form heterodimers with the common β-subunit receptor (CD131), which display a 1000-fold lower affinity for EPO compared with the homodimeric EPOR [3, 67, 68]. EPO binding to EPOR/CD131 engages intracellular pathways overlapping with those of the classical homodimeric EPOR—such as PI3K, MAPK, and STAT5—while also modulating NF-κB activity [62]. The activation of EPOR/CD131 elicits anti-inflammatory effects, tissue repair promotion, and immune homeostasis maintenance, independent of erythropoiesis [62, 70–72]. The relative contribution of homodimeric versus heterodimeric EPOR signalling to the immune system, however, has not been fully elucidated yet.

In CKD, anaemia results primarily from inadequate endogenous EPO production [73] and is further exacerbated by chronic inflammation, which reduces iron availability through cytokine-mediated mechanisms involving TNF-α, IFN-γ, and hepcidin upregulation [74–78]

### Effects of EPO on innate immunity

**Figure 2:**
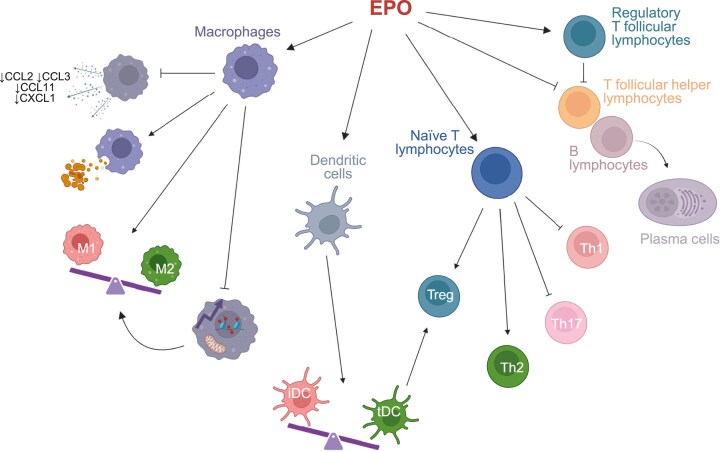
Immunomodulatory pathways of EPO. EPO exerts immunomodulatory effects on both innate and adaptive immune responses. EPO modulates macrophage function by reducing the production of pro-inflammatory chemokines, enhancing phagocytosis of apoptotic cells, promoting polarization towards an anti-inflammatory M2 phenotype, and limiting trained immunity. EPO also inhibits DC maturation, favouring the development of tolerogenic (tDC) rather than immunogenic phenotype (iDC), that promote Treg induction. EPO also affects T-cell differentiation by promoting Treg and Th2 induction, while suppressing pro-inflammatory Th1 and Th17 responses, thereby contributing to an anti-inflammatory response. Additionally, EPO stimulates regulatory T follicular cells and inhibits T_FH_ cell activity. These effects result in an attenuation of B-cell maturation and differentiation into plasma cells. Created using BioRender.

Experimental evidence indicates that EPO exerts significant immunomodulatory effects on both innate and adaptive immune responses, as summarized in Fig. 2.

In macrophages, EPO signalling is predominantly associated with anti-inflammatory effects. *In vitro* and *in vivo* studies have shown that EPO suppresses macrophage production of inflammatory mediators and several chemokines, including CCL2, CCL3, CCL11, and CXCL1, in a dose-dependent manner [79, 80]. Murine models of organ transplantation, kidney injury, colitis, and SLE have confirmed that EPO limits prolonged macrophage infiltration, while facilitating macrophage recruitment to sites of tissue damage to support repair processes [79–85]. In addition, EPO promotes macrophage polarization towards an anti-inflammatory M2 phenotype through the activation of the JAK2/STAT5 pathway downstream the EPOR [81, 86, 87]. Finally, EPO enhances macrophage-mediated clearance of apoptotic cells through PPARγ activation, a mechanism of particular relevance to SLE, where defective apoptotic cell clearance is central to pathogenesis. Consistently, mice with EPOR-deficient macrophages have shown impaired phagocytosis and lupus-like symptoms [88, 89]. Intriguingly, liver tumours that evade immune surveillance produce EPO. Selective EPOR deletion in macrophages prevents tumour development and expansion in mice, indicating that EPO/EPOR signalling in macrophages critically modulates antitumour immune responses. Consistently, EPOR expression in human tumours inversely correlates with immune cell infiltration, supporting a broader role of EPO/EPOR signalling in regulating macrophage-driven immunity across pathological contexts [90]. EPOR is also expressed on DCs [91]. Zhang and colleagues recently demonstrated that EPOR signalling in type 1 conventional dendritic cells (cDC1s) functions as immunologic switch that promotes tolerance by enhancing efferocytosis-associated maturation and inducing Treg. In contrast, cDC1-specific loss of EPOR drives a shift towards an immunogenic phenotype, augmenting antitumour T-cell responses [92].

### Effects of EPO on adaptive immunity

Resting T cells express low levels of EPOR, which is rapidly upregulated upon T-cell receptor (TCR) activation [3, 93]. EPO signalling through the homodimeric EPOR suppresses naïve and memory T-cell proliferation without inducing apoptosis [94, 95]. This effect restricted to activated T cells, as EPO/EPOR signalling induces STAT5 phosphorylation only upon TCR engagement [96].

Upon TCR and costimulatory signalling, T cells produce IL-2, driving clonal expansion via an autocrine loop. EPO/EPOR activation engages Src homology 2-containing inositol phosphatase-1 (SHIP-1), which cross-talks with the β chain of the IL-2 receptor (IL-2Rβ) to inhibit AKT and ERK phosphorylation, thereby limiting T-cell activation and proliferation. SHIP-1 may also dampen proximal TCR signalling, reinforcing EPOR-mediated suppression of T-cell responses [97].

*In vitro*, EPO–EPOR interaction suppresses Th17 differentiation by inhibiting RAR-related orphan receptor C (RORC) expression, even under Th17-polarizing conditions, through reduced SGK1 phosphorylation [98]. *In vivo*, murine models of autoimmune disease, including autoimmune kidney disease, confirmed that EPO limits Th17 induction [98, 99]. EPO also promotes the differentiation of naïve CD4+ T cells into induced Tregsvia TGF-β production by antigen-presenting cells through EPOR/CD131-dependent signalling, involving urokinase-type plasminogen activator-mediated activation of latent TGF-β [94]. We found that a single injection of 10 000 IU of EPO alpha increases functional Treg in stable patients with autoimmune hepatitis, a condition characterized by reduced Treg number and function [100]. Similarly, kidney transplant recipients with erythrocytosis due to increased endogenous EPO exhibit higher Treg levels [101], supporting the relevance of these immunoregulatory effects across clinical settings.

Tregs express EPOR and depend on IL-2 for survival and proliferation. Unlike its inhibitory effect on effector T cells, EPO does not impair Treg function. EPO-induced SHIP-1 activation inhibits IL-2Rβ signalling but spares Tregs by preserving IL-2Rγ/STAT5 signalling and maintaining constitutive AKT suppression via endogenous phosphatases [3, 94].

Finally, EPO inhibits follicular helper T-cell (T_FH_) differentiation and T-cell-dependent B-cell maturation and antibody formation [102].

### Immunomodulating effects of EPO in kidney disease and transplantation

In experimental models of kidney injury, toxin exposure, or hypoxia, exogenous EPO exerts anti-inflammatory and tissue-protective effects [103, 104]. These include reduced TNF-α and IL-1β production, increased IL-10 levels, decreased mononuclear cell chemotactic protein-1 (MCP-1) expression, reduced inflammatory cell infiltration, inhibition of interstitial fibrosis, and preservation of kidney function. Consistently, a prospective cohort study in CKD patients showed that EPO administration at doses used for anaemia correction is associated with increased circulating Tregs [94]. In models of immune-mediated kidney diseases, EPO exerts immunomodulatory and renoprotective effects. In amurine SLE, EPO treatment reduces splenic hyperplasia, proteinuria, and anti-dsDNA antibodies, improving renal histopathology and decreasing glomerular IgG and C3 deposition [105]. These effects are associated with reduced inflammatory cytokine expression in spleen and kidney, and a shift in adaptive immunity towards increased Th2 and Treg cells with concomitant reduction of Th1 and Th17 responses. Consistently, in MRL/lpr mice, endogenous EPO reduces T- and B-cell activation and autoantibody production, suggesting a physiological counter-regulatory role in maintaining immune homeostasis [106, 107]. EPO also upregulates heme oxygenase-1, attenuates oxidative stress, and reduces inflammation and apoptosis in experimental membranous nephropathy [108, 109].

In organ transplantation, EPO has emerged as a regulator of macrophage-driven alloimmune responses. Ischemia–reperfusion injury triggers the release of damage-associated molecular patterns, which rapidly recruit and activate macrophages, promoting inflammation, graft infiltration, and acute rejection. In this context, macrophages can undergo epigenetic and metabolic reprogramming that enhances their responses to subsequent stimuli—a process known as trained immunity—which contributes to accelerated allograft rejection [110–113]. In a murine heart transplant model, macrophage training with the toll-like receptor agonist CpG accelerates graft rejection, whereas EPO both prevents and reverses this process, promoting anti-inflammatory M2 polarization, suppressing proinflammatory cytokine production, and reprogramming transcriptional profiles towards immunoregulation. These effects may translate into improved graft outcomes and prolonged allograft survival, highlighting modulation of trained immunity as a potential therapeutic strategy in transplantation [114].

Consistently, preclinical transplant models demonstrate that EPO prolongs graft survival also through promotion of Treg expansion, limitation of effector T-cell responses, and support of macrophage-mediated apoptotic cell clearance. Importantly, combining EPO with costimulation blockade potently suppresses immune-mediated graft rejection, though further optimization is required to achieve stable immune tolerance [86, 94].

Overall, these findings formed the basis for a prospective phase 1/2 trial (EVEREST; NCT06832189) testing the hypothesis that EPO, in combination with the mechanistic Target of Rapamycin mechanistic Target of Rapamycin (mTOR) inhibitor everolimus, increases Treg and promotes tolerance in liver transplant recipients.

Overall, EPO emerges as an immunomodulatory and renoprotective factor across various kidney conditions, with potential therapeutic applications beyond anaemia management. Large clinical trials have shown increased cardiovascular risk when targeting higher hemoglobin levels [115]. Protumourigenic effects have also been reported. While initially attributed to direct proliferative effects of EPO on cancer cells [116], these may also reflect the immune inhibitory effects of EPO, analogous to other immune-modulating therapies.

## CONCLUSION

Vitamin D and EPO are renal-derived hormones with established roles in mineral metabolism and erythropoiesis, respectively, but accumulating evidence supports broader, partially overlapping effects on immune regulation.

First, both pathways modulate innate and adaptive immunity, influencing immune cell activation and inflammatory balance. This suggests potential relevance across immune-mediated kidney diseases and transplantation, although their effects are context-dependent and not yet fully defined.

Second, current clinical use of vitamin D analogues and EPO is not guided by immunological endpoints. While preclinical data are compelling, clinical evidence for meaningful immunomodulatory benefits remains limited and sometimes inconsistent.

Third, these pathways may represent therapeutic targets, but translation into practice is premature. Rigorous mechanistic studies and well-designed clinical trials are needed to define efficacy, optimal dosing, and patient selection.

Overall, vitamin D and EPO highlight the kidney’s role in systemic immune regulation, but their integration into immunomodulatory strategies in nephrology requires further evidence.

## Data Availability

No new data were generated or analysed in support of this research.
